# Supplementary material for: Cloud BioLinux: pre-configured and on-demand bioinformatics computing for the genomics community
Source: BMC Bioinformatics. 2012 Mar 19;13:42. doi: 10.1186/1471-2105-13-42 (PMC3372431; doi:10.1186/1471-2105-13-42)
Supplement: Additional file 1 — Supplementary 1 Cloud BioLinux software documentation in the form of a mini, self-contained website. Users need to download and uncompress the .zip file, and open through a web browser the "index.html" file available on the main directory. (ZIP 1823 kb). [file 1471-2105-13-42-S1.ZIP › Cloud-BioLinux-Package-Documentation/docs/pedro.html]

Bio-Linux Software Documentation Pages

Back to search form

## pedro

|  |  |
| --- | --- |
| Name | pedro |
| Description | **Pedro** is an application that creates data entry forms based o�n a data model written in a particular style of XML Schema. Users can enter data through the forms to create data files that conform to the schema. They can use controlled vocabularies to mark-up text fields and have the application perform basic validation o�n field data. When they feel they have finished writing a data file, Pedro can tell them if they have left out any required records.  **References:**  Taylor CF, Paton NW, Garwood KL, Kirby PD, Stead DA, Yin Z, Deutsch EW, Selway L, Walker J, Riba-Garcia I, Mohammed S, Deery MJ, Howard JA, Dunkley T, Aebersold R, Kell DB, Lilley KS, Roepstorff P, Yates JR 3rd, Brass A, Brown AJ, Cash P, Gaskell SJ, Hubbard SJ, Oliver SG.  A systematic approach to modeling, capturing, and disseminating proteomics experimental data. Nat Biotechnol. 2003 Mar;21(3):247-54 |
| Homepage | http://pedro.man.ac.uk/ |
| Remote Documentation |  |
